# Variation Analysis of Starch Properties in Tartary Buckwheat and Construction of Near-Infrared Models for Rapid Non-Destructive Detection

**DOI:** 10.3390/plants13152155

**Published:** 2024-08-03

**Authors:** Liwei Zhu, Fei Liu, Qianxi Du, Taoxiong Shi, Jiao Deng, Hongyou Li, Fang Cai, Ziye Meng, Qingfu Chen, Jieqiong Zhang, Juan Huang

**Affiliations:** 1 Research Center of Buckwheat Industry Technology, College of Life Science, Guizhou Normal University, Guiyang 550025, China; 201505005@gznu.edu.cn (L.Z.); 18386012438@gznu.edu.cn (F.L.); 222100100416@gznu.edu.cn (Q.D.); shitaoxiong@gznu.edu.cn (T.S.); ddj613@163.com (J.D.); lihongyouluod@163.com (H.L.); caifang919@gmail.com (F.C.); iorimouse@126.com (Z.M.); cqf1966@163.com (Q.C.); 2 Guizhou Provincial Agricultural Technology Extension Station, Guiyang 550001, China; gzsnjz@126.com

**Keywords:** NIR, resistant starch, total starch, amylose, amylopectin, PLS

## Abstract

Due to the requirements for quality testing and breeding Tartary buckwheat (*Fagopyrum tartaricum* Gaerth), it is necessary to find a method for the rapid detection of starch content in Tartary buckwheat. To obtain samples with a continuously distributed chemical value, stable Tartary buckwheat recombinant inbred lines were used. After scanning the near-infrared spectra of whole grains, we employed conventional methods to analyze the contents of Tartary buckwheat. The results showed that the contents of total starch, amylose, amylopectin, and resistant starch were 532.1–741.5 mg/g, 176.8–280.2 mg/g, 318.8–497.0 mg/g, and 45.1–105.2 mg/g, respectively. The prediction model for the different starch contents in Tartary buckwheat was established using near-infrared spectroscopy (NIRS) in combination with chemometrics. The Kennard–Stone algorithm was used to split the training set and the test set. Six different methods were used to preprocess the spectra in the wavenumber range of 4000–12,000 cm^−1^. The Competitive Adaptive Reweighted Sampling algorithm was then used to extract the characteristic spectra, and the prediction model was built using the partial least squares method. Through a comprehensive analysis of each parameter of the model, the best model for the prediction of each nutrient was determined. The correlation coefficient of calibration (Rc) and the correlation coefficient of prediction (Rp) of the best models for total starch and amylose were greater than 0.95, and the Rc and Rp of the best models for amylopectin and resistant starch were also greater than 0.93. The results showed that the NIRS-based prediction model fulfilled the requirement for the rapid determination of Tartary buckwheat starch, thus providing an effective technical approach for the rapid and non-destructive testing of starch content in the food science and agricultural industry.

## 1. Introduction

As the saying goes, “Grains, buckwheat is king”. *Fagopyrum* Mill is an important multigrain crop divided into two main cultivars: common buckwheat (*Fagopyrum esculentum Moench*) and Tartary buckwheat [1]. *F. tataricum*, also known as Tartary buckwheat, is one of the most important buckwheat species cultivated in China, and is suitable for cultivation in cool climates. Tartary buckwheat grains are notable for their nutritional functional substances, including protein, resistant starch, and flavonoids. The protein content of buckwheat is rich and of high quality, consisting of 19 amino acids, including 8 essential amino acids in appropriate proportions, exceeding the standards set by the Food and Agriculture Organization of the United Nations [2].

The starch content of Tartary buckwheat is about 70%, with a high proportion of resistant starch, far exceeding that of rice and other grains. Resistant starch cannot be directly absorbed and broken down by the human small intestine, providing significant physiological and health benefits, such as maintaining intestinal activity and controlling blood glucose levels [3,4]. The flavonoid content in Tartary buckwheat exceeds 2%, with rutin, quercetin, kaempferol, and hypericin being the primary compounds, with rutin accounting for over 80% of the total flavonoids. These compounds have beneficial effects such as lowering blood sugar, blood pressure, and blood lipids, and possess anti-inflammatory and antibacterial properties [5]. In recent years, the high nutritional value of Tartary buckwheat has driven rapid market demand in the functional food and medicine industries, among others, making it a plant star of the 21st century with substantial market potential and development prospects [1,3,6].

Starch is the main component of Tartary buckwheat grains, accounting for 43.80% to 84.67% of their total weight, and is composed of amylose (11.06–49.24%) and amylopectin (8.97–61.85%) [7]. The quality of food is significantly influenced by the amylose content to amylopectin ratio, which plays a crucial role in yield composition, nutrition, health benefits, and processing quality [3]. Resistant starch, existing in five types (RS1–RS5), refers to starch and its degradation products that cannot be absorbed in the human small intestine. It is a functional component of dietary fiber in foods, helping to control post-meal blood glucose levels, with its yield being proportional to amylose content [8,9]. Therefore, improving the starch content and composition of Tartary buckwheat grains, particularly breeding varieties with high amylose, a high amylopectin ratio, and high resistant starch, is essential for breeding high-yield and high-quality buckwheat. Currently, total starch, amylose, and amylopectin contents are primarily determined by ultraviolet spectrophotometry [10,11,12,13], and resistant starch content is mainly determined by the chromogenic enzymolysis method [14]. Some scientists have developed an asymmetric field-flow separation technique to determine the resistant starch content in potatoes (*Solanum tuberosum* L.) [15]. These methods are laborious, time-consuming, and costly, posing a bottleneck in the genetic research and breeding of high-yield and high-quality Tartary buckwheat.

Near-infrared spectroscopy (NIRS) detection technology offers rapid, accurate, cost-effective, and non-destructive analysis, saving time and cost in sample processing [15]. Once the model is built, the rapid detection of substance content can be achieved through relatively simple instructions and data processing. NIRS has been widely used in food quality control [16], food adulteration [17], the real-time batch process monitoring of medicines [18], and other fields [15]. The near-infrared technique has also been studied in buckwheat [19,20,21,22,23]. For example, Sato et al. used NIR reflectance spectroscopy to analyze moisture, fat, protein, and physiological activity in buckwheat flour for breeding selection, finding that NIR could successfully estimate these contents for simple and rapid breeding selection [20]. Shruti et al. developed an NIRS prediction model for oil, protein, amino acids, and fatty acids in amaranth (*Amaranthus tricolor* L.) and buckwheat, enabling the identification of trait-specific germplasm as potential gene sources and aiding crop improvement programs [21]. Chai et al. found that combinations of NIRS and chemometrics indicated excellent predictive performance and applicability to analyze the adulteration of common buckwheat flour in Tartary buckwheat flour [22]. NIR reflectance spectroscopy has been applied to determine the contents of rutin and D-chiro-inositol in Tartary buckwheat [19].

Previous research highlights two primary near-infrared starch-absorption ranges: 1063–1639 nm and 1834–2175 nm [23], equivalent to wavenumbers of 6101–9407 cm^−1^ and 4598–5453 cm^−1^. Scientists have developed NIR detection models to determine the total starch, amylose, and amylopectin contents of buckwheat, sorghum (*Sorghum bicolor* (L.) Moench), and rice (*Oryza sativa* L.) [24,25,26]. NIR models have also been developed and used to predict resistant starch content and material screening in barley (*Hordeum vulgare* L.) [27], potato [28], rice [29], and sweet potato (*Ipomoea batatas* (L.) Lam.) [30]. However, many studies require spectrum collection after the core has been crushed [20,22], increasing the workload for modeling and making it unsuitable for rare materials resulting from the breeding process. Additionally, NIR spectroscopy for determining resistant starch content in Tartary buckwheat has not yet been reported. 

Diverse and continuous modeling samples are required in the construction of near-infrared models. When Sato et al. constructed a predictive model for the moisture content of buckwheat flour, they used 96 buckwheat varieties from 25 countries to obtain samples with large differences in chemical values [20]. To obtain better modeling results, pure turmeric was mixed with starch in different amounts from 1% to 50%, with the starch varying by 1%, and the created near-infrared model could accurately identify adulterations in turmeric (*Curcuma longa* L.) [17]. Previous studies by our research group have found that the starch content of the recombinant inbred line population has an approximately normal distribution, and the coefficient of variation is between 6% and 18% [10], which is suitable for the construction of near-infrared models. Based on NIRS technology, rapid detection models for total starch, amylose, amylopectin, and resistant starch in Tartary buckwheat have been established, which is of great significance for the quality evaluation of Tartary buckwheat and the development of functional foods. In this study, data were collected from the recombinant inbred line population of Tartary buckwheat to obtain chemical values with a large coefficient of variation and finally obtain a more satisfactory prediction model.

## 2. Results

### 2.1. Assay Results and Variation Analysis of Tartary Buckwheat Starches

As shown in Table 1, the total starch content in Tartary buckwheat ranges from 532.1 mg/g to 741.5 mg/g, the amylose content ranges from 176.8 mg/g to 280.2 mg/g, the amylopectin content ranges from 318.8 mg/g to 497.0 mg/g, and the resistant starch content ranges from 45.1 mg/g to 105.2 mg/g. The degree of dispersion for the four types of starch is relatively large, indicating that the content of these substances in the samples varies significantly, which provides a well-represented dataset.

**Figure 1:**
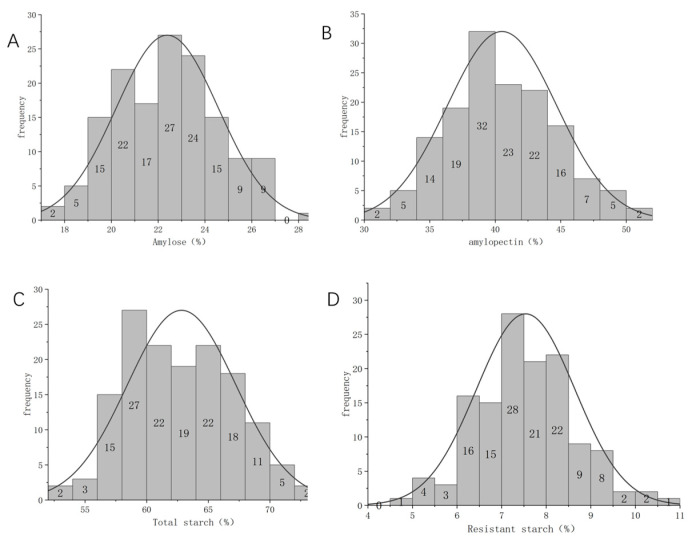
Frequency distribution of starch-related traits in the grains of the Tartary buckwheat RIL population. (**A**) Total starch; (**B**) amylose; (**C**) amylopectin; (**D**) resistant starch.

Based on the frequency distribution analysis of each starch, the chemical values of total starch, amylose, amylopectin, and resistant starch all exhibit an approximately normal distribution, with most values concentrated near the average value (Figure 1A–D). This indicates that the chemical values of the different types of starch in the sample vary only moderately and that there are no extreme outliers.

### 2.2. Establishment of Models

#### Partition of the Sample Set

In this experiment, the Kennard–Stone (KS) algorithm was used to split the dataset. It was found that the model has better predictive ability when the training set and the test set are split in the ratios of 3:1 and 4:1. Table 2 and Table 3 show the proportions of the training set and test set in the modeling process. When the ratio of the samples is 3:1 or 4:1, the specific information of the samples used in the modeling includes the means of the training set and the test set for amylose, amylopectin, total starch, and resistant starch. This ensures that the chemical values of the test set are as close as possible to the range of the training set.

### 2.3. Creation

#### 2.3.1. Modeling Effect When the Ratio of Training Set to Test Set Is 3:1

When the ratio of the training set to the test set changes, the model results vary. In creating the amylose prediction model, six preprocessing methods were applied to the spectra: “Normalization”, “Normalization + MSC”, “Normalization + SNV”, “Normalization + First Derivative”, “Normalization + Second Derivative”, and “Normalization + SG”. The Competitive Adaptive Reweighted Sampling (CARS) algorithm divided the total wavelength into 2307 points, selecting 141–188 points for modeling, corresponding to 6.1% to 8.1% of the total wavelength. These selected spectra showed a high correlation with the chemical values, so the partial least squares (PLS) method was used for modeling. The number of principal components in the model is 13.

As shown in Table 4, among the six preprocessing conditions, only the models created with normalized plus second-derived preprocessing spectra had Rc and Rp values above 0.9 and RPD values above 3.0, indicating good predictive ability. The remaining models all had poor relevant indicators, with RPD values below or just above 1.5, indicating unusable or poor predictive power.

#### 2.3.2. Modeling Effect When the Ratio of Training Set to Test Set Is 4:1

During the creation of amylose prediction models, six pretreatment methods were used to process the spectrum. The CARS algorithm was then employed to select 141–188 wavenumber points from a total of 2307 points, accounting for 6.1–8.1% of the total wavenumber, to narrow the wavenumber range. Following this, the PLS method was used to build the model. When the number of principal components was 13, the model was selected.

As shown in Table 5, the R_c_ and R_p_ of the model constructed using “normalized + derivative” preprocessing spectra are both above 0.96, with RPD values of 4.40 and 3.58, respectively. Among these, the “normalized + first derivative” model is the best, with R_c_ and R_p_ values of 1.00 and 0.97, respectively. Additionally, the RPD values of the other models are greater than 1.5, indicating that they can be used to predict the chemical value of amylose.

**Figure 2:**
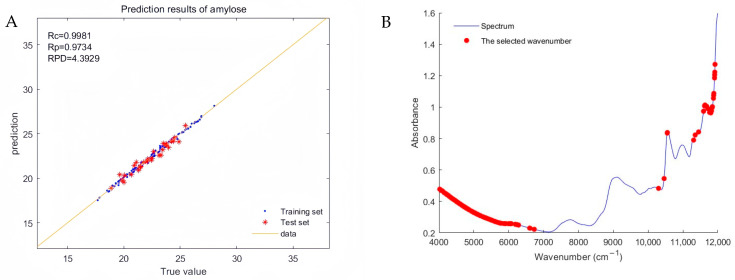
(**A**) Regression diagram of true and predicted amylose values and (**B**) the selected effective wavelengths corresponding to the raw spectrum.

Figure 2A shows the optimal model created using “normalization + second-order derivative” pre-processing spectra when the ratio of the training set to the test set is 4:1, and the regression plot of the true and predicted values of amylose in the model. As seen in the figure, most data points are close to the best-fit line, indicating the high prediction accuracy and reliability of the model. Figure 2B demonstrates that the characteristic spectral points used in the modeling are mainly concentrated in the near-infrared spectral regions of 4000–7000 cm^−1^ and 10,000–12,000 cm^−1^.

### 2.4. Establishment of Amylopectin Prediction Model

#### 2.4.1. Modeling Effect When the Ratio of Training Set to Test Set Is 3:1

In modeling the amylopectin content in buckwheat grains, the spectra underwent preprocessing with six methods, followed by the selection of 141–179 wavelength points out of 2307 using the CARS algorithm, comprising 6.1–7.8% of the total wavelength. The model with 13 principal components was chosen via PLS modeling. Table 6 illustrates the significant variations in prediction effectiveness among the models generated by the six pretreatment methods. The model performance is optimal following “normalization + second derivative” pretreatment, with R_c_ and R_p_ exceeding 0.93 and RPD approaching 2.5, enabling the rapid prediction of amylopectin content in Tartary buckwheat.

#### 2.4.2. Modeling Effect When the Ratio of Training Set to Test Set Is 4:1

When the ratio of the training set to the test set was 4:1, six pretreatment methods were applied to preprocess the spectra accordingly. Subsequently, the CARS algorithm filtered out 129–179 wavenumber points from a total of 2307 points, constituting 5.6–7.8% of the total wavenumber, reducing the spectral dimensionality. The PLS method was then employed to construct the model with 13 principal components.

Table 7 highlights that the most effective model utilized the “normalization + second derivative” pretreatment spectrum, achieving R_c_ and R_p_ values of 1.00 and 0.90, respectively. This model enables the rapid and accurate prediction of amylopectin content in buckwheat.

**Figure 3:**
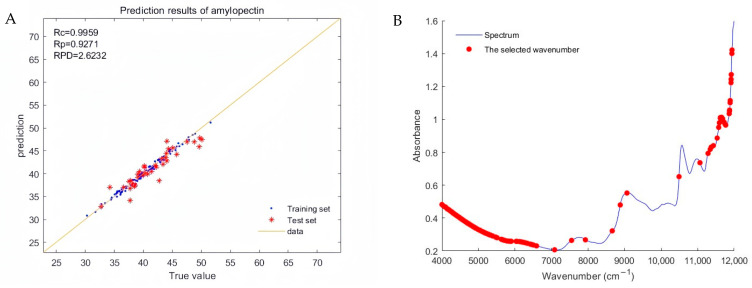
(**A**) Regression plot of true and predicted values of amylopectin, and (**B**) selected effective wavelengths corresponding to the raw spectrum.

Figure 3A depicts the regression plot of the optimal model for amylopectin generated using the “normalization + second derivative” pretreatment spectra. As shown in the figure, the data points of the true and predicted values closely align around the best-fit line, demonstrating a high correlation. The model exhibits strong prediction performance, suitable for the rapid determination of amylopectin content in whole Tartary buckwheat grains.

As shown in Figure 3B, the characteristic spectral points used in the modeling are primarily concentrated in the near-infrared spectral regions of 4000–7000 cm^−1^ and 11,000–12,000 cm^−1^. Additionally, some spectral points distributed in the near-infrared spectral region of 7000–9000 cm^−1^ were also utilized.

### 2.5. Establishment of Total Starch Prediction Model

#### 2.5.1. Modeling Effect with a Training Set to Test Set Ratio of 3:1

Initially, six different pretreatment methods were applied to process the spectrum. Subsequently, the CARS algorithm filtered out 112–155 wavenumber points from 2307 waves, accounting for 4.9–6.7% of the total wavenumber, thereby enhancing wavenumber utilization efficiency. The PLS method was employed for modeling, utilizing 13 principal components.

As illustrated in Table 8, the best model was achieved using “normalization + second derivative” pretreatment spectroscopy. The model exhibited R_c_ and R_p_ values of 1.00 and 0.95, respectively, with an RPD of 3.38, indicating a strong predictive capability for total starch content in Tartary buckwheat. Conversely, the RMSEP and RPD for the “normalized + MSC” and “normalized + SG” preprocessing spectra were less favorable. However, the Rc and Rp values for the other models exceeded 0.95 and 0.85, respectively, with RPD values surpassing 1.5, all indicating robust predictive performance.

#### 2.5.2. Modeling Effect When the Ratio of Training Set to Test Set Is 4:1

After applying six different pretreatments, 112–155 characteristic wavenumber points were selected, constituting 4.9–6.7% of the total wavenumber. The model with 13 principal components was chosen. As shown in Table 9, the optimal model resulted from “normalization + derivative” spectrum processing, achieving R_c_ and R_p_ values both above 0.94 and an RPD of about 3.0, indicating superior predictive capability. However, models generated from the “normalization + MSC” and “normalization + SG” pretreatments of the spectra yielded RPD values of 0.31 and 1.36, respectively, rendering them unusable at less than 1.5. The RPD of the remaining models exceeded 1.5, indicating acceptable predictive performance, albeit moderate.

**Figure 4:**
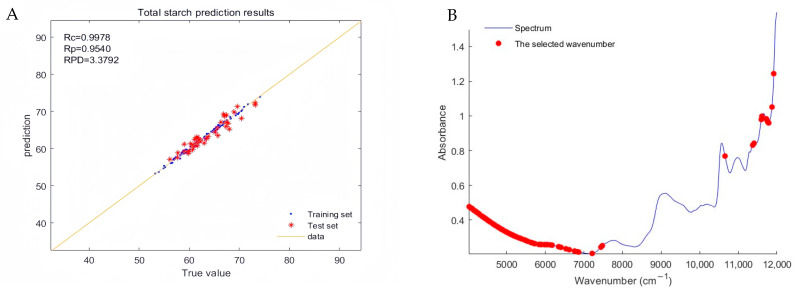
(**A**) Regression plot of total starch’s true and predicted values and (**B**) the selected effective wavelengths corresponding to the raw spectrum.

Figure 4A depicts the regression plot of the optimal total starch model constructed using “normalized + second derivative” pretreatment spectra. As observed in the figure, the data points of the training set are nearly all closely aligned with the best-fit line, while those of the test set exhibit slight dispersion but mostly cluster around the fit line, indicating the model’s overall strong predictive capability.

Figure 4B shows that the characteristic spectral points used in the modeling are primarily concentrated in the near-infrared spectral ranges of 4000–7500 cm^−1^ and 11,000–12,000 cm^−1^.

### 2.6. Establishment of Resistant Starch Prediction Model

#### 2.6.1. Modeling Effect When the Ratio of Training Set to Test Set Is 3:1

The CARS algorithm filtered out 106–129 wavenumber points from 2307 waves, accounting for 4.6% to 5.6% of the total wavenumber, thereby improving wavenumber utilization efficiency. The PLS method was used for modeling, with 13 principal components selected for the model. As shown in Table 10, the best model was obtained using “Normalization + First derivative” to preprocess the spectrum. The R_c_ and R_p_ of the model were 1.00 and 0.94, respectively, and RPD was 2.76, indicating good predictive ability for accurately determining the content of resistant starch in Tartary buckwheat.

Except for the model constructed using the “Normalization + Second derivative” pre-processing spectrum, which had a small RMSEP and an RPD greater than 1.5, thus showing acceptable performance, the RPD values of the other models were less than 1.5, rendering those models unsuitable for use.

#### 2.6.2. Modeling Effect When the Ratio of Training Set to Test Set Is 4:1

The CARS algorithm was employed to select varying numbers of characteristic wavenumber points from 2307 wavenumber points to construct the model, with 13 principal components chosen for modeling. As depicted in Table 11, the best model was developed using the “normalized + SNV” pretreatment spectrum, achieving R_c_ and R_p_ values of 1.00 and 0.93, respectively, and an RPD of 2.71, indicating excellent predictive ability for swiftly and accurately assessing the resistant starch content in Tartary buckwheat.

Furthermore, the RMSEP of the model constructed using the “normalization + first derivative” preprocessing spectrum was considerable, with an RPD greater than 1.5, demonstrating effective performance. However, the RPD of the other models was less than 1.5, rendering those models unsuitable for use.

**Figure 5:**
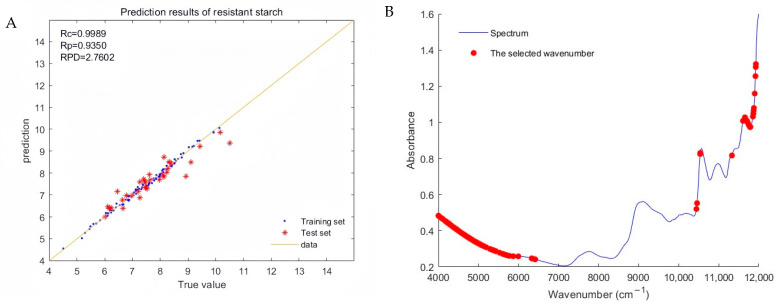
(**A**) Regression plot of resistant starch’s true and predicted values and (**B**) the selected effective wavelengths corresponding to the raw spectrum.

Figure 5A depicts the regression plot of the optimal total strength model created using the “normalization + first derivative” preprocessing spectra. As observed in the figure, the data points of the training set are closely clustered around the best-fit line, while those of the test set exhibit slight scattering, yet most remain near the fit line, demonstrating the model’s strong predictability.

Figure 5B illustrates that the characteristic spectral points utilized in the modeling are primarily concentrated in the near-infrared spectral ranges of 4000–6000 cm^−1^ and 10,500–12,000 cm^−1^. Additionally, some spectral points distributed in the near-infrared spectral range of 6000–6500 cm^−1^ were also utilized.

## 3. Discussion

In plant breeding research, e.g., QTL mapping, general populations are large, and the determination of chemical values is particularly time-consuming and laborious. Scientists use RIL population modeling to predict chemical values, perform QTL mapping, and achieve good results [31,32,33]. In this study, near-infrared models were successfully constructed to predict the amylose, amylopectin, total starch, and resistant starch levels of Tartary buckwheat. The Rc and Rp values of the best model were both above 0.93, meaning that this inbred line can be used to achieve the high-quality production, breeding, and QTL mapping of Tartary buckwheat. However, modeling RIL populations also has disadvantages. Zhang et al. compared the differences between RIL population modeling and natural population modeling and found that the model created by the natural population had a better predictive effect on exotic samples. They hypothesized that this could be related to the narrower range of chemical values in RIL populations [32]. The construction of the model is influenced by various factors, with sample representativeness and diversity being among the most critical. A broader range of chemical values in the sample enhances the applicability of the created model. Through the extensive analysis of starch in Tartary buckwheat germplasm, significant variation in starch content among varieties was observed: total starch ranged from 604.9 mg/g to 779.8 mg/g, amylose from 115.9 mg/g to 283.0 mg/g, amylopectin from 377.1 mg/g to 591.5 mg/g, and resistant starch from 47.4 mg/g to 225.3 mg/g [1,9,34,35,36,37]. In contrast to earlier studies, the total starch content measured in this research using RIL populations ranged from 532.1 mg/g to 741.5 mg/g, amylose from 176.8 mg/g to 280.2 mg/g, amylopectin from 318.8 mg/g to 497.0 mg/g, and resistant starch from 45.1 mg/g to 105.2 mg/g. Therefore, the predicted results of samples with chemical values within the range of RILs will be more accurate. To extend the scope of the model, we need to add samples from outside the inbred lineage group to the modeling samples. 

The prediction results of the near-infrared model for the sample were closely related to the chemical values of the sample used in the modeling. Resistant starch content in this study (45.1 mg/g to 105.2 mg/g) fell outside the range of prior measurements [1], likely due to differences in detection methods influenced by cooking and other processing procedures. The study by Fu et al. found that the resistant starch content in ‘Sichuan Buckwheat No. 1’ Tartary buckwheat measured directly by the Goñi method was 4.74%. After improving the determination method, the resistant starch content could reach 13.38% [34], which is relatively close to the starch content we measured. Zheng et al. used the Englyst method to determine the resistant starch content in black Tartary buckwheat. Using different enzyme dosages and ratios, they determined a resistant starch content between 20% and 33% [1], which is significantly higher than the value we measured. Therefore, the resistant starch content predicted by the model established here is based on the chemical measurements conducted in this paper.

In the modeling process, sample set distribution influences model creation. Common partitioning methods include random partitioning, the KS algorithm, and the SPXY (Sample Set Partitioning based on joint x-y distance) algorithm. The KS algorithm, reliant on sample similarity, balances subsets post-partitioning, preventing over-bias towards specific sample types and enhancing model generalization across different subsets [38]. SPXY, an extension of the KS algorithm, comprehensively considers sample concentration and spectral distance for sample screening. Wang et al. applied the SPXY and KS algorithms to partition sample sets and model soybean meal nutrients, finding KS to be superior for water and protein modeling [39]. In this study, the KS algorithm effectively partitioned samples for modeling, reaffirming its utility in model construction.

Prior to model creation, spectrum preprocessing is essential to eliminate errors caused by noise and other factors during spectrum scanning. Each preprocessing method yields different effects, necessitating the exploration and comparison of various methods [38]. Normalization primarily removes irrelevant variables’ influence on data, such as instrument sensitivity, sample size, and optical path length, to highlight the signal [40]. The standard normal variate (SNV) eliminates effects of particle size, surface scattering, and light path variations on diffuse reflected light [41]. Multiplicative scatter correction (MSC) addresses scattering effects resulting from non-uniform particle distribution and size [42,43]. Savitzky–Golay (SG) filtering aids in signal denoising, data smoothing, and feature extraction [44]. Derivatives effectively eliminate baseline and background noise, enhancing resolution and sensitivity [45]. The model construction effects in this study demonstrate superior prediction using “normalization + derivative” spectrum preprocessing. Scaling data post-normalization to a specific range mitigates dimensional differences among features, enhancing model training efficiency and stability. While this algorithmic processing may induce baseline drift [45], derivative processing effectively mitigates baseline drift and superposition effects, significantly improving model prediction effectiveness. The SNV primarily normalizes grain size disparity, contributing effectively to whole-grain spectrum modeling.

To address challenges such as low absorption intensity, large spectral bandwidth, NIR spectra overlap, high information redundancy, and the strong collinearity of the whole spectrum [46], variable selection methods are essential to extract useful wavelength variables. The CARS algorithm identifies key variables for modeling from thousands of wavenumbers, improving model predictability and reducing complexity [47]. The CARS compression of characteristic bands for each soil type to less than 16% of the total wavenumber substantially reduces soil hyperspectral variable dimensions and computational complexity, thereby enhancing calibration model predictability [48]. Previous research highlights two primary near-infrared starch-absorption ranges: 1063–1639 nm and 1834–2175 nm [23], equivalent to wavenumber of 6101–9407 cm^−1^ and 4598–5453 cm^−1^. Zhang et al. observed significant differences in sorghum variety absorbance at 932 and 978 nm while assessing sorghum amylose and amylopectin using near-infrared techniques [25]. Our study found that optimal model wavenumber points are concentrated in the 4000–7000 cm^−1^ range, with additional points in the 10,000–12,000 cm^−1^ range, consistent with prior findings, underscoring CARS’s suitability for enhancing near-infrared modeling and spectrum utilization.

Recent advancements have achieved notable success in developing models using near-infrared spectroscopy combined with chemometric methods for the rapid determination of plant starch content [49]. However, studies indicate that the spectral modeling of crushed samples often outperforms whole-grain spectrum modeling [20,22]. While modeling crushed samples minimally impacts the assessment of grain content as a raw food material [50], it is less suitable for NIR technology applications in breeding, particularly in relation to predicting substance content in valuable seed materials. Recent research has significantly enhanced spectrum processing effectiveness, improving spectrum utilization efficiency. Some researchers have begun utilizing complete grain scan spectra for modeling, extracting more spectral information from samples while simplifying modeling workflows [51]. Addressing the need for the rapid and non-destructive determination of amylose and amylopectin in sorghum breeding, Peiris et al. successfully developed a near-infrared model to directly detect linear chain and amylopectin in grains, achieving a correlation coefficient of about 0.8 [52]. In this study, spectral processing methods were further optimized, refining characteristic spectral modeling and successfully establishing a near-infrared model capable of predicting total starch, amylose, amylopectin, and resistant starch content in Tartary buckwheat grains. The test series of the best model exhibited correlation coefficients above 0.93, indicating improved spectrum utilization efficiency and feasibility for predicting nutrients in whole-grain crops. Promisingly, near-infrared hyperspectral imaging technology has enabled models to detect starch content in individual corn kernels [53,54]. In this study, the CARS algorithm directly extracted useful variables from full Tartary buckwheat grain spectra. This approach simplifies work processes, eliminates the need for hulling or crushing Tartary buckwheat grains during modeling, and enhances model practicality for breeding purposes, improving breeding efficiency and reducing costs. Future steps will explore using near-infrared or hyperspectral imaging of individual Tartary buckwheat grains to develop rapid, non-destructive predictive models for various nutrient components, facilitating early-stage progeny screening in breeding efforts.

## 4. Materials and Methods

### 4.1. Experimental Materials and Spectrum Acquisition

In this experiment, a stable Tartary buckwheat RIL population was utilized as the study subject. The male parent of this population was ‘*Jinbuckwheat No. 2*’ and the female parent was ‘*Millet buckwheat*’, resulting in a total of 175 lines. Harvested seeds were initially dried in an electric blast drying oven (Model MGL-125B, Taisite Instrument, Tianjin, China) at 60 °C for 2 to 3 days. Subsequently, using an MPA Fourier transform NIR spectrometer (Model MPA, Bruker Corporation, Karlsruhe, Germany), NIR spectra of the Tartary buckwheat seeds were recorded employing the diffuse reflectance method. Each sample underwent 64 single scans with a resolution of 4 cm^−1^ over the scanning range of 4000–12,000 cm^−1^. Three scans were conducted per sample, and the spectral average was calculated to obtain the final modeling spectrum. Additionally, some dried grains were crushed and hulled using a high-speed grinder (Model FW100, Taisite Instrument, Tianjin, China), followed by passing through an 80-mesh sieve to determine their chemical values.

### 4.2. Determination of Starch-Related Traits

#### 4.2.1. Determination of Amylose and Amylopectin Content

Amylose and amylopectin levels were determined using the double-standard two-wavelength colorimetry method established by our research team [10]. Buckwheat seed powder stored at low temperature was taken after crushing (Section 4.1); approximately 0.1 g was weighed, and 10 mL of anhydrous ethanol was added. The mixture was placed in a water bath (Model DK98-2, Taisite Instrument, Tianjin, China) at 80 °C for 30 min, removed, and rapidly cooled. After centrifugation (Model D2012 plus, Dalong Xingchuang Experimental Instruments (Beijing) Co., Ltd., Beijing, China) at 7000 rpm for 5 min, the supernatant was discarded. Subsequently, 10 mL of 1 mol/L KOH solution was added, and the mixture was placed in a water bath at 100 °C for 15 min. After rapid cooling, it was shaken evenly, and 1 mL was extracted and added to 10 mL of distilled water. From this mixture, 200 μL was taken, to which 1 mL of distilled water was added and mixed well, and the pH was adjusted to approximately 3.0. Then, 20 μL of iodine reagent was added, followed by 2 mL of distilled water. The solution was thoroughly shaken, and distilled water was used as a blank control. The solution was left to react for 25 min in the dark. Absorbance values of the reaction solution at 597 nm, 480 nm, 541 nm, and 700 nm were determined using an ultraviolet spectrophotometer (Model T6-1650E, Puxi General Instrument Co., Ltd., Beijing, China), with three replicates per sample.

#### 4.2.2. Determination of Total Starch Content

Total starch content was determined based on the content of amylose and amylopectin, as described in Section 4.2.1.

Total starch content (%) = amylose content + amylopectin content.

#### 4.2.3. Determination of Resistant Starch Content

Resistant starch content was determined according to the method for determining resistant starch content in maize [55]:

(1) Determination of reduced sugar content: Initially, 0.1 g of anhydrous glucose was accurately weighed into a small beaker and dissolved in distilled water. The solution was transferred to a 100 mL volumetric flask, adjusted to volume, and thoroughly shaken to obtain a glucose standard solution with a concentration of 1 mg/mL. Seven test tubes (25 mL each) were prepared by adding 0.0, 0.2, 0.4, 0.6, 0.8, 1.0, and 1.2 mL of the glucose standard solution, respectively, followed by the addition of 2.0, 1.8, 1.6, 1.4, 1.2, 1.0, and 0.8 mL of distilled water. Subsequently, 1.5 mL of 3,5-dinitrosalicylic acid reagent was added to each tube, mixed thoroughly, and heated in a boiling water bath for 5 min. After cooling rapidly to room temperature, 25 mL of distilled water was added and thoroughly mixed. The absorbance values were measured at a wavelength of 540 nm using distilled water as a blank control. A standard curve was constructed using the number of glucose milligrams as the x-coordinate and the absorbance value as the y-coordinate [56].
(2) Determination of resistant starch content: The Goñi method [57] was slightly improved for determining resistant starch. Initially, a sample of 2.000 g was accurately weighed and placed in a 150 mL triangular bottle. A HCl-KCl buffer solution (pH 2.0–4.0) was added, followed by 0.1 mL of pepsin (1 g/100 mL), and the bottle was swirled in a water bath at 60 °C for 1 h to remove protein from the sample.

Next, the material was removed and cooled to room temperature, and the pH was adjusted to 5.4 using 0.1 mol/L HCl and 0.1 mol/L NaOH. Then, 2 mL of 1% heat-stable amylase was added, and the sample was swirled in a water bath at 90 °C for 1.5 h. This step hydrolyzed starch into glucoamylase and dextrin into small molecules.

After cooling the sample to room temperature, the pH was adjusted to 4.1–4.3, and 1 mL of 0.01% glucoamylase was added to the water bath at 40 °C. The dextrin was fully hydrolyzed to monosaccharides by shaking for 12 h.

Following this, the mixture was precipitated with four times the volume of anhydrous ethanol and centrifuged for 20 min at 4000 rpm; then, the supernatant was discarded. The precipitate was washed three times with 80% ethanol to completely remove the glucose. The precipitate was then dissolved and precipitated with 5 mL of 2 mol/L KOH solution, and the pH was adjusted to 4.1–4.3. Subsequently, 1 mL of 0.01% glucoamylase was added, and the mixture was hydrolyzed at 40 °C for 45 min. Afterward, it was heated in a boiling water bath for 5 min and centrifuged again for 20 min at 4000 rpm; then, the supernatant was collected. This process was repeated twice with 10 mL of distilled water. The combined supernatant was adjusted to a final volume of 100 mL.

Finally, the content of reduced sugars in the supernatant was determined using the method described in (1), and the amount of resistant starch was calculated by multiplying the data by 0.9 [58].

### 4.3. Data Processing and Model Evaluation

#### 4.3.1. Data Processing Software

In this study, the systematic analysis of total starch, amylose, amylopectin, resistant starch, and other data, as well as the creation of corresponding images, were performed using Origin 2022 software. Matlab R2023b software was employed to develop the models.

#### 4.3.2. Evaluation of Models

The quality of a model can be assessed by parameters such as the coefficients of determination for the training set and the test set (R_c_, R_p_), root mean square error (RMSE), and the coefficient of prediction (RPD) [59]. RMSE represents the predictive ability of the model, with smaller values indicating better performance. R_c_ and R_p_ denote the degree of agreement between predicted and actual values for the test set and training set, respectively, with values closer to 1 indicating better agreement. RPD reflects the accuracy and robustness of the model [60]. Specific guidelines for RPD are as follows: RPD < 1.5 indicates poor predictive ability and unsuitability for prediction; 1.5 ≤ RPD < 2.5 indicates moderate predictive ability and potential suitability for prediction; RPD ≥ 3.0 indicates strong predictive ability and suitability for quantitative analysis in practical applications [61].

## 5. Conclusions

Considering that the conventional methods for assessing Tartary buckwheat’s starch in laboratories are typically time-consuming and expensive, requiring specialized testing instruments, the nondestructive measurement of chemical components in Tartary buckwheat with considerable accuracy is now feasible. In this work, the KS algorithm was used to extract characteristic spectra, and appropriate spectral preprocessing methods were used to develop NIR models. A series of low-cost, environmentally friendly, non-destructive, and efficient models were established to predict the starch content in Tartary buckwheat grains. The R_c_ and R_p_ of the best models for total starch and amylose were greater than 0.95, and the R_c_ and R_p_ of the best models for amylopectin and resistant starch were also greater than 0.93. The prediction results of these models demonstrated high accuracy, good stability, and reliability, suggesting their potential to replace chemical methods for determining the starch components in Tartary buckwheat grains. The near-infrared model developed in this study is grounded in the starch chemical value determination method utilized herein.

## Figures and Tables

**Table 1:** Statistical analysis results of Tartary buckwheat starch substances detected using UV spectrophotometry.

Traits	Sample Size	Range (mg/g)	Mean Value (mg/g)	Skewness	Kurtosis
Total starch	147	532.1–741.5	628.3	0.244	−0.553
Amylose	146	176.8–280.2	223.9	0.211	−0.543
Amylopectin	147	318.8–497.0	388.8	0.097	−0.912
Resistant starch	132	45.1–105.2	75.4	0.093	0.162

**Table 2:** Sample data used in modeling when the ratio of training set to test set is 3:1.

Traits	Sample Set	Sample Size	Range (mg/g)	Mean Value (mg/g)
Amylose	Training set	117	176.8–280.2	224.1
	Test set	29	188.8–254.6	223.2
Amylopectin	Training set	118	303.1–516.1	401.8
	Test set	29	365.9–500.8	418.8
Total starch	Training set	118	532.1–741.5	625.5
	Test set	29	576.2–732.3	639.8
Resistant starch	Training set	105	45.1–101.7	75.2
	Test set	26	60.2–94.3	94.3

**Table 3:** Sample data used in modeling when the ratio of training set to test set is 4:1.

Traits	Sample Set	Sample Size	Range (mg/g)	Mean Value (mg/g)
Amylose	Training set	110	176.8–280.2	223.6
	Test set	36	188.9–265.8	226.4
Amylopectin	Training set	110	303.1–516.1	401.6
	Test set	37	327.4–500.8	415.8
Total starch	Training set	110	532.1–741.5	624.8
	Test set	37	561.5–732.3	639.0
Resistant starch	Training set	98	45.1–101.3	74.9
	Test set	33	60.2–101.7	76.0

**Table 4:** Influence of pretreatment methods on amylose model when the ratio of training set to test set is 3:1.

Pretreatment Method	R_c_	R_p_	RMSECV (mg/g)	RMSEP (mg/g)	RPD	WLP
Normalization	0.98	0.83	4.0	11.1	1.69	141
Normalization + MSC	0.99	0.83	3.2	41.0	0.46	188
Normalization + SNV	0.98	0.85	4.4	9.8	1.93	155
Normalization + First derivative	1.00	0.91	1.5	7.9	2.38	148
Normalization + Second derivative	1.00	0.95	1.6	6.2	3.06	141
Normalization + SG	0.99	0.84	3.8	10.5	1.80	171

Note: MSC: multiplicative scatter correction; SNV: standard normal variate transform; SG: Savitzky–Golay smoothing filter; R_c_: correlation coefficient of calibration; R_p_: correlation coefficient of prediction; RMSECV: root mean square error of cross-validation; RMSEP: root mean square error of prediction; RPD: ratio of the standard error of prediction to the standard deviation of the reference values; WLP: wavelength points; the same below.

**Table 5:** Influence of pretreatment methods on amylose model when the ratio of training set to test set is 4:1.

Pretreatment Method	R_c_	R_p_	RMSECV (mg/g)	RMSEP (mg/g)	RPD	WLP
Normalization	0.99	0.89	3.8	8.1	2.14	141
Normalization + MSC	0.99	0.92	3.9	9.1	1.92	188
Normalization + SNV	0.98	0.90	4.2	7.9	2.21	155
Normalization + First derivative	1.00	0.97	1.4	4.0	4.40	148
Normalization + Second derivative	1.00	0.96	1.5	4.9	3.58	141
Normalization + SG	0.99	0.93	3.9	6.2	2.83	171

**Table 6:** Influence of pretreatment methods on amylopectin model when the ratio of training set to test set is 3:1.

Pretreatment Method	R_c_	R_p_	RMSECV (mg/g)	RMSEP (mg/g)	RPD	WLP
Normalization	0.98	0.80	9.1	25.9	1.64	155
Normalization + MSC	0.98	0.81	8.0	24.7	1.72	141
Normalization + SNV	0.98	0.85	9.1	23.7	1.79	179
Normalization + First derivative	1.00	0.90	3.6	18.2	2.34	135
Normalization + Second derivative	1.00	0.93	3.7	16.2	2.62	155
Normalization + SG	0.96	0.79	11.1	27.1	1.57	129

**Table 7:** Influence of pretreatment methods on amylopectin model when the ratio of training set to test set is 4:1.

Pretreatment Method	R_c_	R_p_	RMSECV (mg/g)	RMSEP (mg/g)	RPD	WLP
Normalization	0.97	0.77	9.4	24.9	1.53	155
Normalization + MSC	0.98	0.86	7.9	27.3	1.39	171
Normalization + SNV	0.98	0.86	9.3	21.0	1.81	179
Normalization + First derivative	1.00	0.86	3.8	19.4	1.95	135
Normalization + Second derivative	1.00	0.90	3.7	17.5	2.17	155
Normalization + SG	0.96	0.73	11.2	28.1	1.35	129

**Table 8:** The influence of pretreatment methods on the total starch model when the ratio of training set to test set is 3:1.

Pretreatment Method	R_c_	R_p_	RMSECV (mg/g)	RMSEP (mg/g)	RPD	WLP
Normalization	0.98	0.82	9.3	25.3	1.71	148
Normalization + MSC	0.98	0.84	8.5	42.5	1.02	141
Normalization + SNV	0.96	0.87	11.6	21.2	2.04	112
Normalization + First derivative	1.00	0.91	2.8	18.1	2.40	155
Normalization + Second derivative	1.00	0.95	2.9	12.8	3.38	148
Normalization + SG	0.97	0.74	10.0	32.9	1.31	155

**Table 9:** Influence of pretreatment methods on the total starch model when the ratio of training set to test set was 4:1.

Pretreatment Method	R_c_	R_p_	RMSECV (mg/g)	RMSEP (mg/g)	RPD	WLP
Normalization	0.98	0.84	9.1	21.9	1.85	148
Normalization + MSC	0.99	0.89	7.3	131.1	0.31	141
Normalization + SNV	0.96	0.83	11.7	23.1	1.75	112
Normalization + First derivative	1.00	0.95	3.1	13.8	2.93	155
Normalization + Second derivative	1.00	0.95	2.9	12.7	3.20	148
Normalization + SG	0.97	0.73	10.1	29.7	1.36	155

**Table 10:** Effects of pretreatment methods on resistant starch model when the ratio of training set to test set is 3:1.

Pretreatment Method	R_c_	R_p_	RMSECV (mg/g)	RMSEP (mg/g)	RPD	WLP
Normalization	0.96	0.57	3.3	8.8	1.10	123
Normalization + MSC	0.98	0.70	2.4	19.2	0.51	129
Normalization + SNV	0.96	0.62	3.0	8.1	1.21	106
Normalization + First derivative	1.00	0.94	0.5	3.5	2.76	129
Normalization + Second derivative	0.99	0.79	1.2	6.3	1.54	112
Normalization + SG	0.96	0.55	2.9	8.9	1.09	117

**Table 11:** Effects of pretreatment methods on resistant starch model when the ratio of training set to test set is 4:1.

Pretreatment Method	R_c_	R_p_	RMSECV (mg/g)	RMSEP (mg/g)	RPD	WLP
Normalization	0.94	0.76	3.9	7.5	1.47	102
Normalization + MSC	0.96	0.61	3.1	7.5	1.18	106
Normalization + SNV	1.00	0.93	0.5	3.3	2.71	129
Normalization + First derivative	0.99	0.80	1.1	5.7	1.56	112
Normalization + Second derivative	0.97	0.72	2.9	7.1	1.24	117
Normalization + SG	0.96	0.67	3.0	13.9	0.64	117

## Data Availability

The data presented in this study are available upon request from the corresponding author.
